# A Prospective Study on Functional Outcome of Surgical Management of Acetabular Fractures

**DOI:** 10.7759/cureus.82243

**Published:** 2025-04-14

**Authors:** Sudev Raghunathan, Ravikumar Biradar, Ashok Nayak, Vijaykumar Patil

**Affiliations:** 1 Orthopaedics, Bijapur Lingayat District Educational Association (BLDE) (Deemed to be University) Shri B. M. Patil Medical College, Hospital and Research Centre, Vijayapura, IND; 2 Orthopaedic Surgery, Bijapur Lingayat District Educational Association (BLDE) (Deemed to be University) Shri B. M. Patil Medical College, Hospital and Research Centre, Vijayapura, IND

**Keywords:** acetabular fractures, functional outcome, hip fractures, kocher-langenbeck approach, modified merle d’aubigne score, modified stoppa approach, surgical management, trauma

## Abstract

Background

Acetabular fractures represent complex injuries that present significant management challenges. This prospective study evaluates the functional outcomes of surgical management of acetabular fractures at a tertiary care center and identifies factors influencing these outcomes.

Methods

Thirty-one patients with acetabular fractures who underwent surgical management between 2023 and 2025 were enrolled in this prospective study. Fractures were classified according to the Judet-Letournel system. Functional outcomes were assessed using the Merle d’Aubigne score at presentation and six weeks, three months, and six months post-surgery. Demographic data, fracture characteristics, surgical approaches, complications, and associated injuries were documented. Statistical analysis was performed to identify factors associated with functional outcomes.

Results

The study cohort was comprised predominantly of young adult males (71%), with road traffic accidents being the primary mechanism of injury (71%). Posterior column fractures and anterior column fractures with associated pubic rami fractures (25.8% each) were the most common patterns, followed by posterior wall fractures (19.4%). Associated posterior hip dislocations were present in 25.8% of cases. The Kocher-Langenbeck approach was most frequently employed (45.2%), followed by the modified Stoppa approach (32.3%). Functional assessment revealed progressive improvement, with all patients demonstrating poor scores at presentation, progressing to 51.6% excellent and 48.4% moderate outcomes by six months. Complications were observed in 19.4% of patients, with hip stiffness being the most common (12.90%). Age, fracture pattern, associated dislocation, surgical approach, and post-surgery follow-up significantly influenced functional outcomes, while the presence of complications also showed a significant association (p=0.03).

Conclusion

Surgical management of acetabular fractures yields favorable functional outcomes with progressive improvement over time. Most demographic and fracture characteristics significantly influenced outcomes, and the results support the efficacy of tailored surgical approach selection and meticulous technique. The findings highlight the importance of extended rehabilitation and careful management of complications to optimize functional recovery.

## Introduction

Acetabular fractures represent one of the most challenging injuries in orthopedic trauma surgery, requiring extensive expertise in both diagnosis and management. These complex injuries, often resulting from high-energy trauma, have significant implications for patient mobility and quality of life. The intricate anatomy of the acetabulum, combined with its crucial role in weight-bearing and hip joint function, makes accurate reduction and fixation paramount for achieving optimal outcomes [1].

The understanding and treatment of acetabular fractures have evolved significantly since the pioneering work of Judet and Letournel in the 1960s. Their classification system, which remains the gold standard today, provided a systematic approach to analyzing these injuries and planning surgical intervention. Despite technological advances in imaging and surgical techniques, acetabular fractures continue to present substantial challenges to orthopedic surgeons, with reported positive outcomes varying significantly across different studies and treatment approaches [2].

The incidence of acetabular fractures has shown a bimodal distribution, with peaks in young adults following high-energy trauma and in elderly patients after low-energy falls. Recent epidemiological studies indicate a growing trend in geriatric acetabular fractures, attributed to increased life expectancy and higher activity levels among older adults. This demographic shift has introduced new challenges in management strategies, as elderly patients often present with compromised bone quality and multiple comorbidities [3].

The decision-making process in the management of acetabular fractures involves careful consideration of multiple factors, including fracture pattern, patient age, bone quality, associated injuries, and the timing of intervention. The goal of surgical treatment is to achieve “anatomical reduction of the articular surface and stable fixation, allowing early mobilization and reducing the risk of post-traumatic arthritis”. However, the complex three-dimensional anatomy of the acetabulum and its surrounding neurovascular structures makes surgical intervention technically demanding [4].

Advanced imaging techniques have revolutionized the preoperative planning process. While conventional radiographs remain fundamental, computed tomography (CT) with three-dimensional reconstruction has become indispensable for understanding fracture morphology and planning surgical approaches. These imaging modalities help surgeons better appreciate the fracture configuration, degree of comminution, and presence of intra-articular fragments, all of which influence the choice of surgical approach and fixation strategy [5].

The timing of surgery represents a critical factor in outcome determination. The traditional window of opportunity for optimal surgical intervention has been established as within 5-7 days post-injury, allowing for patient stabilization and soft tissue recovery while avoiding the complications associated with delayed surgery. However, recent studies have challenged this conventional wisdom, suggesting that outcomes may be acceptable even with delayed intervention in carefully selected cases where patients with hip dislocation who in spite of undergoing reduction at the primary center still presented with dislocation at the time of presentation and the mean time to operative surgery was 11.3 days (ranging from 1 to 42 days) [6].

Surgical approaches to the acetabulum have also evolved significantly. The choice between anterior, posterior, or combined approaches depends on fracture pattern, surgeon expertise, and patient factors. The development of minimally invasive techniques and specialized instruments has expanded the surgical options available, particularly for simple fracture patterns or elderly patients who may not tolerate extensive surgical exposure. However, the role of these newer techniques continues to be defined through ongoing research and clinical experience [7].

Post-operative rehabilitation plays a crucial role in determining functional outcomes. The development of standardized protocols, incorporating early mobilization and progressive weight-bearing, has contributed to improved results. However, the optimal timing and progression of rehabilitation remain subjects of debate, particularly in complex fracture patterns or in patients with compromised bone quality [8].

The assessment of functional outcomes following acetabular fracture surgery presents unique challenges. Various scoring systems have been developed to evaluate postoperative results, including the Harris Hip Score, Merle d’Aubigné Score, and patient-reported outcome measures. These tools help quantify functional recovery and facilitate comparison between different treatment strategies and study populations [9].

Complications following acetabular fracture surgery can significantly impact functional outcomes. “These include post-traumatic arthritis, heterotopic ossification, avascular necrosis of the femoral head, and infection.” Understanding the risk factors for these complications and developing strategies for their prevention and management remain active areas of research. The increasing use of specialized surgical approaches and prophylactic measures has helped reduce complication rates, though they remain a significant concern [10].

The present study aims to evaluate the functional outcomes of surgically managed acetabular fractures, considering various factors that may influence results. Through careful documentation of patient characteristics, surgical techniques, and postoperative outcomes, we hope to contribute to the existing knowledge base and potentially identify factors associated with improved functional results.

## Materials and methods

This is a prospective study conducted in the Department of Orthopedics at BLDE (Deemed to be University) Shri B.M. Patil Medical College, Hospital and Research Centre, Vijayapura, Karnataka, which was conducted from March 2023 to March 2025, and a total of 31 patients who had sustained acetabular fractures were included in this study.

Inclusion criteria included all elementary fractures of acetabular fractures, including anterior column, anterior wall fractures, posterior wall, posterior column fracture, transverse fractures, and also associated fracture patterns of bicolumnar fracture along with a willingness to participate in regular follow-ups at regular intervals.

Exclusion criteria included patients less than 18 years old and more than 70 years old. Patients with an undisplaced acetabular fracture and patients with an ipsilateral femur fracture who didn’t have regular follow-ups for at least six months and were unfit for surgery.

The patients were interviewed for the collection of necessary information using the pre-tested, semi-structured questionnaire method (Merle D'Aubigne Scoring System) described in Table 1. Patient evaluation began with a detailed clinical examination and thorough history taking. Radiological investigations included X-ray PBHS (pelvis with both hip joints) with an anterior-posterior view, obturator oblique and iliac oblique views, and CT pelvis with 3D reconstruction. Laboratory investigations were extensive, covering hematological, biochemical, and serological parameters.

**Table 1:** Merle D'Aubigne score The Merle D'Aubigne scale is used for the functional outcome of the hip [11]. Scores from the three criteria are added and tallied together, and the score ranges from 3 to 18, where: Very great improvement: 12 or more, Great improvement: 7 to 11, Fair improvement: 3 to 7, Failure: less than 3

Criteria			
Pain	Mobility	Range of motion	Points
None	Normal	95-100%	6
Slight or intermittent	No cane but slight limp	80-94%	5
After walking but resolves	Long-distance with cane or crutch	70-79%	4
Moderately severe, but patients are able to walk	Limited even with support	60-69%	3
Severe, prevents walking	Very limited	50-59%	2
Severe, even at night	Unable to walk	Less than 50%	1

**Figure 1:**
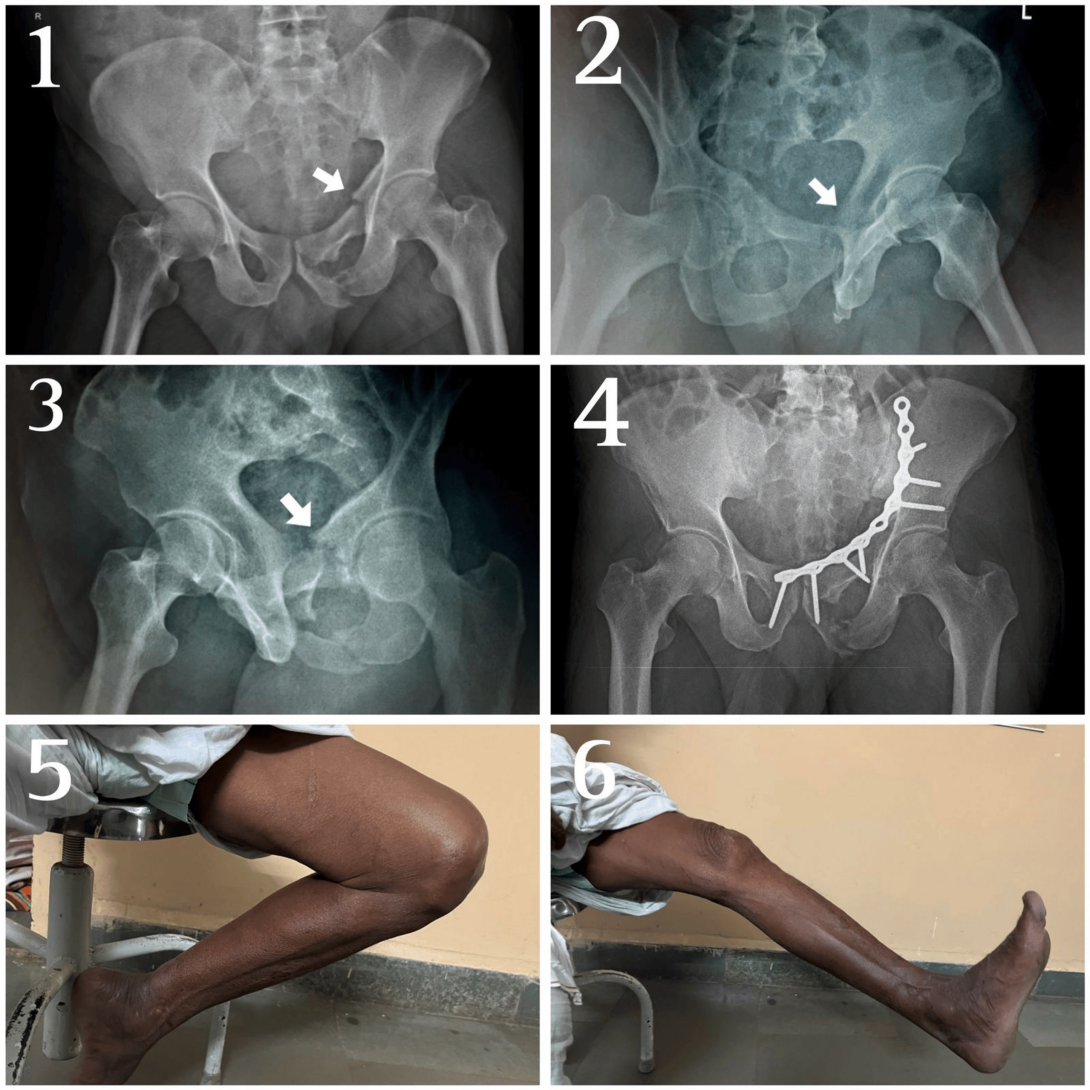
Pre- and postoperative X-rays of patient with anterior column fracture using modified Stoppa approach Pre-operative radiographs—first: Anteroposterior (AP), second: Iliac oblique (IO), and third: Obturator oblique (OO) views with arrows showing the fracture. Fourth: Immediate postoperative radiographs; fifth and sixth: Clinical photographs of patients at six-month follow-up

**Figure 2:**
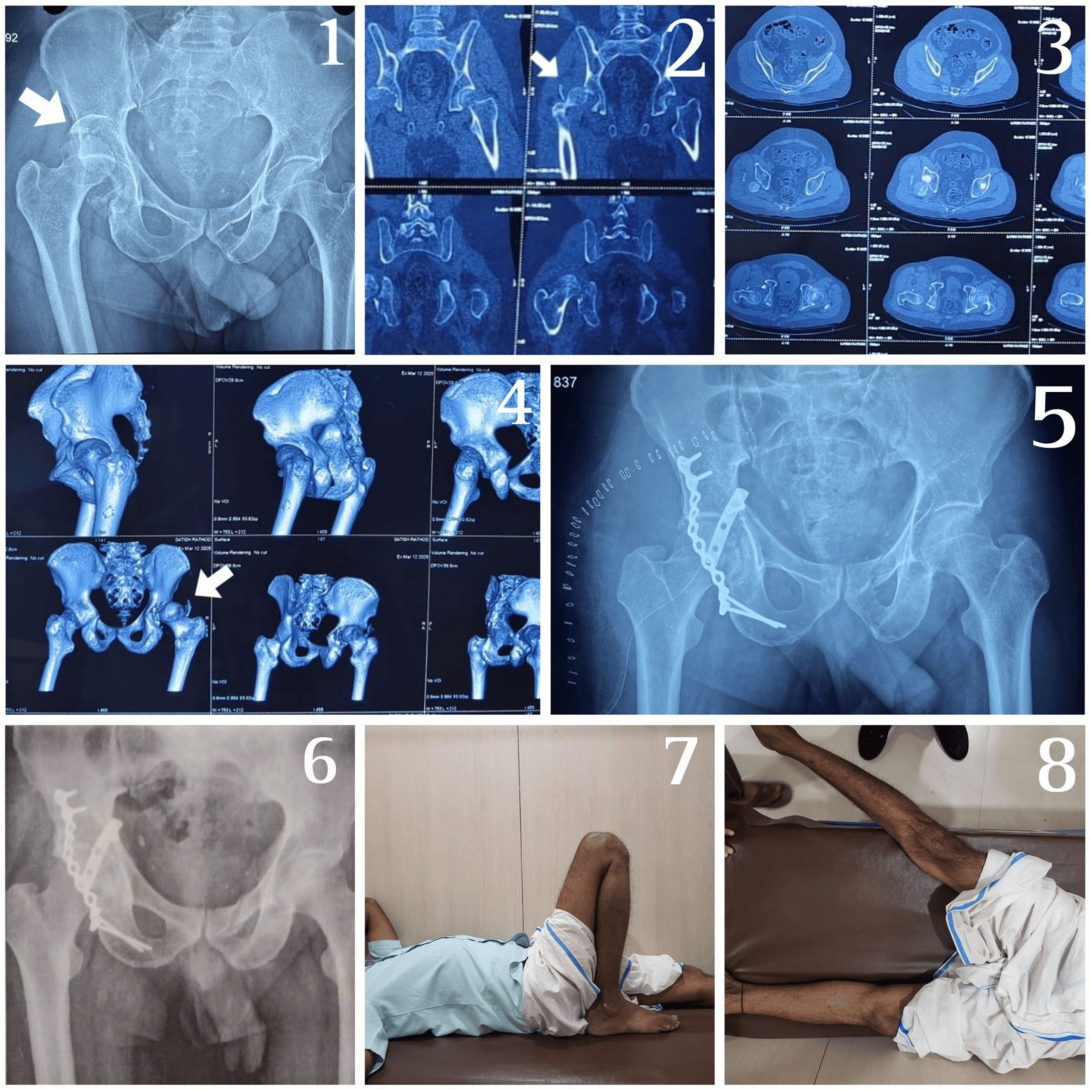
Pre- and postoperative radiographs of a patient with a posterior column fracture using the Kocher-Langenbeck approach First: Preoperative X-ray of the pelvis with both hip joints showing posterior hip dislocation with posterior acetabular wall fracture, which was reduced using the modified Allis method; second, third, and fourth: Computed tomography (CT) images—coronal, axial, and 3D reconstruction cuts, respectively, with fracture fragment reduced; fifth: Immediate postoperative X-ray of the pelvis with both hip joints; sixth: Postoperative X-ray at six-month follow-up; seventh and eighth: Hip movements postoperatively at six months showing excellent results.

The surgical treatment approach was carefully planned, utilizing different surgical approaches based on fracture characteristics. Surgical techniques included a modified Stoppa approach with a lateral window for anterior column fractures [12] as depicted in Figure 1, an ilio-inguinal approach, and a Kocher-Langenbeck approach for posterior column fractures [13] as depicted in Figure 2. Anesthesia methods varied, including spinal, epidural, or general anesthesia, selected based on individual patient requirements. If there was any associated posterior dislocation of the hip, the reduction was achieved before surgical management by a modified Allis method [14].

Postoperative management was meticulously structured. Reduction achievement was confirmed through postoperative radiographs of the pelvis. Patient mobilization was initially restricted to toe-touching weight bearing for the first three months. After fracture consolidation, total weight bearing was allowed under the guidance of a physiotherapist. Follow-up was systematically planned with assessments conducted at six weeks, three months, and six months post-surgery. The radiological evaluation involved an X-ray of the pelvis with both hips in anteroposterior views. Functional outcome was assessed using the Merle d'Aubigne scoring system, providing a comprehensive evaluation of patient recovery and surgical intervention effectiveness. 

Data was entered in an Excel sheet (Redmond, USA) and analyzed using the IBM Corp. Released 2011. IBM SPSS Statistics for Windows, Version 20.0. Armonk, NY: IBM Corp. Descriptive statistics were calculated for all variables, including means, standard deviations, medians, and ranges for continuous data, and frequencies and percentages for categorical data. Continuous variables were expressed as means ± standard deviations, while categorical variables were presented as frequencies and percentages. Paired t-tests and chi-square tests were used to compare preoperative and postoperative continuous variables. Statistical significance was set at p < 0.05.

## Results

In our study population of 31 patients, the majority of patients, 19 (61.3%), were in the younger age group of 20-40 years, while eight (25.8%) were in the middle age group of 41-60 years, and only four (12.9%) were in the older age group of 61-80 years, as depicted in Table 2.

**Table 2:** Distribution of patients according to age The data has been represented as frequency of acetabular fractures in the different age groups and percentage of the population (N,%), p-value is considered significant at <0.05.

Age (in years)	Frequency (Number)	Percentage
20-40	19	61.3%
41-60	8	25.8%
61-80	4	12.9%
Total	31	100%

The gender distribution of patients revealed a significant male predominance, with 22 males (71%) compared to only nine females (29%), as depicted in Table 3. 

**Table 3:** Distribution of patients according to gender The data has been represented as frequency of acetabular fractures for different genders, percentage of the population (N,%), p-value is considered significant at <0.05.

Gender	Frequency (Number)	Percentage
Female	9	29%
Male	22	71%
Total	31	100%

Road traffic accidents (RTA) were the predominant cause, accounting for 22 (71%) of cases, while falls from height contributed to nine (29%) of the injuries, as depicted in Table 4. 

**Table 4:** Distribution of patients according to mode of injury The data has been represented as frequency of acetabular fractures from different modes of injury and percentage of the population (N, %). A p-value is considered significant if it is < 0.05.

Mode of injury	Frequency (Number)	Percentage
Fall from height	9	29%
RTA	22	71%
Total	31	100%

In our study, the distribution of associated injuries, the majority of patients, 28 (90.3%), did not have any associated injuries. Bladder injury was present in two (6.5%) cases, and hemoperitoneum occurred in one (3.2%) patient, as depicted in Table 5.

**Table 5:** Distribution of patients according to associated injuries The data has been represented as distribution of patients according to associated injuries and percentage of the population (N,%), p-value is considered significant at <0.05.

Associated injuries	Frequency	Percentage
Nil	28	90.3%
Bladder injury	2	6.5%
Hemoperitoneum	1	3.2%
Total	31	100%

The Kocher-Langenbeck approach was most commonly employed (14 [45.2%]), followed by the modified Stoppa approach (10 [32.3%]). Less frequently used approaches included combined Kocher-Langenbeck with modified Stoppa (3 [9.7%]), ilioinguinal approach (2 [6.5%]), modified Stoppa with lateral window (1 [3.2%]), and percutaneous pinning (1 [3.2%]), as depicted in Table 6.

**Table 6:** Distribution of patients according to approach The data has been represented as frequency of acetabular fractures according to the surgical approach used, percentage of the population (N,%), p-value is considered significant at <0.05.

Approach	Frequency	Percentage
Ileoinguinal	2	6.5%
Kocher langenbeck	14	45.2%
Kocher Langenbeck+modified stoppa	3	9.7%
Modified stoppa	10	32.3%
Modified stoppa+lateral window	1	3.2%
Percutaneous pinning	1	3.2%
Total	31	100%

In the analyses of the relationship of the Merle d’Aubigne score at different points in time at the time of presentation, six weeks post-operatively, three months post-operatively, and six months post-operatively. The modified Merle d’Aubigne scoring (scores 3-18) mean values at presentation were 5.00 (SD- 1.211), at six weeks postoperatively were 6.71 (SD- 1.637), at three months postoperatively were 9.39 (SD- 1.476), and at six months postoperatively was 12.45 (1.670) and gave a statistically significant p-value of 0.001 as depicted in Table 7.

**Table 7:** Association of Merle D’Aubigne score with progression of time The data has been represented as Merle D'Aubigne scores at different points of follow-up, where the mean Merle D'Aubigne score at particular intervals of time, +/- standard deviation (mean +/- SD), with statistical analysis using Friedman's test value and p-value, is considered significant at <0.05.

Merle D’Aubigne Score	Mean	SD	Friedman's test	p-value
Presentation	5.00	1.211	92.416	0.001
6 Weeks	6.71	1.637		
3 Months	9.39	1.476		
6 Months	12.45	1.670		

In the analyses, the relationship between age and functional outcomes at six months. Among patients with excellent outcomes (scores 13-18), 81.3% (13 out of 16) were young adults aged 20-40 years, while 18.7% (3 out of 16) were middle-aged patients between 41-60 years, and 0% were elderly patients aged 61-80 years. For patients with moderate outcomes (scores 10-12), the age distribution was more evenly spread: A total of 40% (6 out of 15) were aged 20-40 years, 33.3% (5 out of 15) were aged 41-60 years, and 26.7% (4 out of 15) were aged 61-80 years and gave a statistically significant p-value of 0.02 as depicted in Table 8.

**Table 8:** Association of Merle d'Aubigne score at six months with age The data has been represented as Merle D'Aubigne scores with the age of the patient six months post-surgery and the number of patients in each group (moderate and excellent) in percentage (N, %), where statistical analysis with chi-square value has been evaluated with a significant p-value of <0.05.

Age (in years)	Merle d'Aubigne score		Chi square value	p-value
	Moderate (10-12)	Excellent (13-18)		
20-40	6 (40%)	13 (81.3%)	7.05	0.02
41-60	5(33.3%)	3 (18.7%)		
61-80	4 (26.7%)	0		
Total	15 (100%)	16 (100%)		

In the analysis, the impact of associated hip dislocation on functional outcomes. Among patients with excellent outcomes (scores 13-18), 93.8% (15 out of 16) had no associated dislocation, while only 6.2% (1 out of 16) had posterior dislocation. In contrast, among patients with moderate outcomes (scores 10-12), 53.3% (8 out of 15) had no associated dislocation, and 46.7% (7 out of 15) had posterior dislocation with a statistically significant p-value of 0.01, as depicted in Table 9.

**Table 9:** Association of Merle d'Aubigne score at six months with associated dislocation The data has been represented as Merle D'Aubigne scores with any associated dislocation of the hip six months post-surgery with the number of patients in each group (moderate and excellent) and in percentage (N, %), where statistical analysis with chi-square value has been evaluated with a significant p-value of < 0.05.

Associated dislocation	Merle d'Aubigne score		Chi square value	p-value
	Moderate (10-12)	Excellent (13-18)		
Nil	8 (53.3%)	15 (93.2%)	6.6	0.01
Posterior	7 (46.7%)	1 (6.2%)		
Total	15 (100%)	16 (100%)		

In this study, Patients who had no complications were more likely to have excellent outcomes, with this association approaching statistical significance (p=0.03) as depicted in Table 10.

**Table 10:** Association of Merle d'Aubigne score at six months with complications The data has been represented as Merle D'Aubigne scores with any complications six months post-surgery with the number of patients in each group (moderate and excellent) and in percentage (N, %), where statistical analysis with chi-square value has been evaluated with a significant p-value of < 0.05.

Complications	Merle d'Aubigne score		Chi square value (p-value)	p- value
	Moderate (10-12)	Excellent (13-18)		
Nil	10 (66.7%)	14 (87.5%)	0.875	0.6456
Hip stiffness	3(20%)	1 (6.25%)		
Surgical wound infection	1 (6.7%)	0		
Surgical wound infection+hip stiffness	1 (6.7%)	1 (6.25%)		
Total	15 (100%)	16 (100%)		

Posterior column fractures and anterior column fractures with associated pubic rami fractures were the most common (8 [25.8%] each), followed by posterior wall fractures (6 [19.4%]). Anterior column and posterior column combined fractures accounted for four (12.8%) of cases along with approaches taken as depicted in Table 11.

**Table 11:** Distribution of patients according to fracture with approach used

Fracture	Frequency	Percentage	Approach
Anterior column	2	6.5%	Modified stoppa/Percutaneous pinning
Anterior column + posterior column	4	12.9%	Modified Stoppa/ Modified Stoppa + Kocher langenbeck
Anterior column + superior and inferior pubic rami	8	25.8%	Modified stoppa/Ileoinguinal approach
Posterior column	8	25.8%	Kocher Langenbeck
Posterior column + superior and inferior pubic rami	1	3.2%	Modified Stoppa + Kocher langenbeck
Posterior column + posterior wall	2	6.5%	Kocher Langenbeck
Posterior wall	6	19.4%	Kocher Langenbeck
Total	31	100%	

## Discussion

The higher prevalence in younger populations can be attributed to their greater participation in high-risk activities and occupations. This demographic pattern is particularly significant as it highlights the substantial socioeconomic impact of these injuries, affecting individuals in their prime productive years. Letournel's classic series reported 75% male patients, while a multicenter study by the German Pelvic Trauma Registry documented 69% male patients among 2405 acetabular fractures [15]. This gender disparity likely reflects higher male involvement in road traffic accidents, occupational hazards, and high-energy recreational activities. The high proportion of RTA-related acetabular fractures in our study underscores the need for enhanced road safety measures, particularly in developing countries where increasing motorization has not been matched by adequate safety infrastructure. This distribution reflects the predominance of posterior column and posterior wall fractures in our series, for which the Kocher-Langenbeck approach provides optimal exposure. Our preference for the modified Stoppa approach over the traditional ilioinguinal approach for the anterior column and associated fractures aligns with evolving trends in acetabular surgery.

Our study documented diverse fracture patterns according to the Judet-Letournel classification. This distribution partially diverges from patterns reported in other series where posterior wall fractures are most common. Analysis of functional outcomes based on surgical approach revealed interesting patterns; differences did reach statistical significance (p=0.02). Notably, all patients who underwent combined Kocher-Langenbeck with modified Stoppa approaches achieved excellent outcomes. This finding supports the principle that complex acetabular fractures often require extensive exposure through combined approaches to achieve anatomical reduction. Our results are in line with a biomechanical study conducted by Sawaguchi et al., where they used a transverse acetabular fracture specimen in a cadaver; they demonstrated that a stable fracture fixation could only be achieved with a reconstruction plate fixing both columns [16]. Tekin SB et al. reported superior visualization of the quadrilateral plate and reduced surgical morbidity with the modified Stoppa approach compared to the ilioinguinal approach [17]. 

The presence of associated dislocation significantly impacted the functional outcomes at six months in our study (p=0.01). Among patients with associated dislocations, seven out of 15 (46.7%) achieved moderate outcomes, and one out of 16 (6.2%) achieved excellent outcomes. This showed a trend toward better outcomes in patients without dislocations, which did reach statistical significance. This finding aligns with several published studies that have identified associated hip dislocation as a negative prognostic factor. Briffa et al. reported that concomitant hip dislocation increased the risk of poor outcomes by 2.1-fold, primarily due to higher rates of osteonecrosis, chondrolysis, and heterotopic ossification [6]. Similarly, Letournel found that 85% of patients with acetabular fractures without dislocations achieved good to excellent results, compared to 76% of those with associated dislocations [4]. The timing of dislocation reduction is critical in influencing outcomes. Sahin et al. demonstrated that reduction performed within 12 hours significantly improved outcomes compared to delayed reduction [18].

The observation that no patient achieved excellent outcomes before three months and the majority required six months to reach their maximal functional potential, this temporal progression illustrates the prolonged recovery trajectory following acetabular fracture surgery and highlights the importance of patient counseling regarding recovery expectations and the necessity for extended rehabilitation.

Several factors may have contributed to the relatively high proportion of excellent outcomes in our series. First, the predominance of younger patients (19(61.3%) aged 20-40 years) with better healing potential and fewer comorbidities likely influenced overall outcomes positively. Second, the adherence to surgical timing principles, with most procedures performed within the optimal window of 5-10 days, may have facilitated a reduction in quality. Mears et al. demonstrated that surgeries performed within 14 days of injury yielded significantly better outcomes than delayed procedures [5]. Similarly, Thunuguntla R et al. reported that internal fixation of acetabular fractures leads to good outcomes in the majority of patients, emphasizing that early surgical intervention and experienced management are prime factors in achieving favorable results [19]. Finally, the implementation of standardized rehabilitation protocols with early mobilization likely enhanced functional recovery.

It is noteworthy that by six months, all patients had progressed beyond the poor category, suggesting that surgical intervention, regardless of fracture complexity, offers significant functional improvement over the natural history of acetabular fractures. Tile's natural history study of untreated displaced acetabular fractures reported that 80% of patients had persistent poor function and pain, highlighting the transformative impact of appropriate surgical management [20].

The presence of associated urological injuries, particularly bladder trauma, in 6.5% of our patients highlights the importance of comprehensive evaluation in acetabular fractures. The proximity of the bladder and urethra to the anterior acetabular columns and pubic rami necessitates high clinical suspicion for urological injuries, especially in fractures involving the anterior column and pubic rami. Hak et al. reported urological injuries in 8% of pelvic and acetabular fractures, emphasizing the need for multidisciplinary management [21].

Limitations of our study

Several limitations of our study warrant acknowledgment. First, the sample size of 31 patients and being a single-center study, while providing valuable insights, limited the statistical power to detect significant associations between various factors and outcomes. Subgroup analyses, particularly regarding fracture patterns and surgical approaches, would benefit from larger patient cohorts.

Second, the follow-up period of six months, while sufficient to document early functional recovery, may not capture late complications such as post-traumatic arthritis, avascular necrosis, and heterotopic ossification.

Lastly, our reliance on the Merle d’Aubigne score, while providing standardized functional assessment, does not capture patient-reported outcomes regarding quality of life, return to work, and subjective satisfaction. The integration of validated patient-reported outcome measures would provide a more comprehensive assessment of surgical success from the patient’s perspective.

## Conclusions

Acetabular fractures represent complex injuries that predominantly affect young, productive individuals and require meticulous surgical management to restore function. By six months post-surgery, all patients in our cohort had progressed beyond poor functional scores, with most achieving excellent and moderate outcomes as measured by the Merle d’Aubigne score. This progressive improvement over time highlights the importance of patient counseling regarding recovery expectations and the necessity for extended rehabilitation support. The predominance of young adult males in our study and the high proportion of road traffic accidents as the mechanism of injury underscore the significant socioeconomic impact of these fractures and emphasize the need for enhanced prevention strategies. The diversity of fracture patterns encountered, with posterior column fractures and anterior column fractures with associated pubic rami fractures being the most common, reflects the heterogeneous nature of these injuries and reinforces the importance of individualized surgical planning. Our findings support the principle that anatomical reduction and stable fixation, rather than the inherent complexity of the fracture, are the primary determinants of outcome.

The selection of surgical approach should be guided by the fracture pattern, with the Kocher-Langenbeck approach being particularly effective for posterior column and wall fractures, and the modified Stoppa approach offering excellent access to anterior column and quadrilateral plate fractures. Combined approaches may be necessary for complex fracture patterns to achieve optimal reduction. The relatively low complication rate in our series is encouraging and highlights the importance of meticulous surgical technique, appropriate perioperative care, and systematic rehabilitation protocols. The absence of catastrophic complications such as deep infection, significant neurovascular injury, or implant failure speaks to the efficacy of contemporary management principles. In conclusion, surgical management of acetabular fractures delivers predictable and favorable functional outcomes when executed with careful preoperative planning, appropriate approach selection, precise reduction techniques, and structured rehabilitation. Continued refinement of surgical approaches, fixation methods, and rehabilitation protocols will further enhance outcomes for these challenging injuries. Long-term studies will be valuable to assess the durability of functional results and the incidence of post-traumatic arthritis, which remains a concern even with optimal initial management.
